# ‘Name It to Tame It’: Dementia Diagnostic Procedure in Austrian Care Facilities for People With Intellectual Disabilities. An Interview Study

**DOI:** 10.1111/jir.70085

**Published:** 2026-02-02

**Authors:** Annalisa La Face, Dominik Pendl, Barbara Gasteiger‐Klicpera

**Affiliations:** ^1^ University of Graz, Institute of Education Research and Teacher Education, Inclusive Education Unit Graz Austria; ^2^ CERI Center for Empirical Research in Inclusion Graz Austria; ^3^ University College of Teacher Education Styria, Institute for Early Childhood and Primary Teacher Education Graz Austria

**Keywords:** dementia, diagnosis, formal caregivers, intellectual disability, interview study

## Abstract

**Background:**

Despite the growing relevance of dementia in people with intellectual disabilities, there are currently no national recommendations in Austria to inform diagnostic protocols within care facilities. In order to gain a state‐of‐the‐art understanding of the issue, the aim of this study was to map out the process currently followed by formal caregivers when they suspect a person in their care has dementia.

**Method:**

We conducted 30 interviews with formal caregivers working in three care facilities for people with intellectual disabilities in Styria, an Austrian province. The interviews were transcribed and analysed using structured qualitative content analysis.

**Results:**

In order to notice signs of early dementia and start the diagnostic process, a long‐term relationship between formal caregivers and the person with intellectual disabilities appears to be crucial. Standardised observational instruments for monitoring changes were used by only three formal caregivers. In 19 out of 30 cases, no diagnostic assessment was carried out, even though dementia was suspected.

**Conclusions:**

To uphold the right to health for older adults with intellectual disabilities, policies and recommendations must be established in Austria to ensure timely and accurate dementia diagnoses. This requires the introduction of standardised observational and documentation tools, clear diagnostic pathways and specialist diagnostic centres.

## 1 Background

Over the past decades, improvements in the health and living conditions of people with intellectual disabilities in many Western industrialised societies have led to a steady increase in their life expectancy (Dieckmann et al. 2015; Dolan et al. 2019; Putnam et al. 2021). In particular, the life expectancy of people with Down syndrome has more than doubled in the last 70 years (Alzheimer's Association 2025). This demographic change has occurred more recently in countries with a history of Nazi regime rule, such as Germany (Dieckmann et al. 2015) and Austria (Haveman and Stöppler 2020). Here, due to the mass murder of children and adults with intellectual disabilities during the Second World War (‘Aktion T4’; Gabriel 2016), today's population includes the very first individuals with intellectual disabilities who could reach retirement age (Dieckmann et al. 2015).

Nowadays, 41% of residents in disability services in Austria are over 50 years old (Schachner et al. 2022). However, due to the relative newness of this demographic phenomenon, designing services for older people with intellectual disabilities has not yet been prioritised in professional discussions (Denninger 2020; Köhncke 2009). This has led to them being described as the ‘invisible elderly’ (Frewer‐Graumann and Schäper 2015; Schachner et al. 2022). In fact, in Austria, services for people with intellectual disabilities and services for the elderly remain distinct (Schachner et al. 2022). It follows that age‐related diseases, such as dementia, are a relatively new topic in disability care settings in this country, yet one of growing relevance (Pendl et al. 2024).

Dementia is indeed one of the age‐related health conditions that are more prevalent in individuals with intellectual disabilities and Down syndrome than in the general population. This is due to genetic factors in people with Down syndrome (Lott and Head 2019). Regarding individuals with intellectual disabilities without Down syndrome, dementia is presumably related to pre‐existing cognitive impairments, poorer physical health and higher rates of comorbidities (Takenoshita et al. 2020). Nevertheless, in Austria, dementia in this population is often underdiagnosed or diagnosed far too late (Schachner et al. 2022).

Despite the World Health Organization's (WHO) recommending already in 2012 to develop national guidelines on ‘diagnosis, assessment and treatment, as well as quality long‐term care’ (WHO 2012, 62), there is currently no nationally defined protocol for screening and diagnosing dementia in adults with intellectual disabilities (Schachner et al. 2022). The Austrian social welfare system is split into nine different regional legislations, and the national Dementia Strategy, ‘Gut Leben mit Demenz’ (‘Living well with dementia’), acknowledges only a marginal focus on older adults with intellectual disabilities. Moreover, Austria lacks specialist diagnostic services with expertise in dementia and intellectual disability (Schachner et al. 2022), where neuropsychological test batteries specifically designed for this population are routinely administered (e.g., the ‘DTIM’ [Dementia Test for People with Intellectual Disabilities]; Wagner and Weber 2021). In fact, because of the high heterogeneity within this population, most tests for the general population are not suitable for people with intellectual disabilities due to floor effects, especially for persons with severe/profound intellectual disability (Dekker et al. 2021).

Considering the absence of screening possibilities and standardised diagnostic procedures in Austria, the most effective method to identify early dementia symptoms is through caregiver observation (Janicki et al. 2022; McKenzie et al. 2018). The long‐term relationships and frequency of contact entailed in caregiving put caregivers in an ideal position to notice any change in the state of health or in the adaptive behaviour of the persons in their charge, such as behavioural changes, loss of ability or the onset of emotional problems (Bishop et al. 2015). For this reason, people living with their families are diagnosed with dementia earlier than their peers living in institutions (Sinai et al. 2017). In Austria, according to a systematic review commissioned from the Federal Ministry of Social Affairs, Health, Care and Consumer Protection, only an average of 36% of people with intellectual disabilities over the age of 40 still live with their parents (Griebler et al. 2021). Most older adults with intellectual disabilities live in community‐based residential services, the primary support structure for this population in Austria at the moment (Schachner et al. 2022). Therefore, to allow an early dementia diagnosis, the duty of ongoing observation should be taken on by professional caregivers. However, the topic of dementia is currently not legally anchored in the curricula of training institutions for care professions in social services (e.g., in the province of Styria; see Steiermärkisches Sozialbetreuungsberufegesetz 2008/2022; Fessl 2020). As a result, formal caregivers are often ill‐prepared to recognise the early signs of dementia (Pendl et al. 2024).

Since an early diagnosis has a significant impact on the well‐being of the individuals (Dekker et al. 2021), efforts to address the issue are necessary. In the absence of a nationally defined protocol for dementia screening in adults with intellectual disabilities in Austria, the present study aimed at investigating current practices within Austrian care facilities—from the caregivers' initial awareness of subtle or ambiguous changes to the formal diagnosis of dementia, after other potential causes of decline have been ruled out (hearing/vision loss, hypothyroidism, medication intoxication, vitamin deficiencies and depression; Moran et al. 2013).

To comprehensively map out the entire process, the study was guided by the three‐level model proposed by Holst et al. (2018). According to this model, the first level in the process of identifying suspected dementia is formal caregivers, as they play a key role in recognising initial signs. At the second level, internal resources, such as colleagues or facility managers, are involved in discussing and evaluating such cases. The third level of the process involves the engagement of external professionals, including medical doctors, psychologists or specialised services, in order to conduct a formal diagnostic assessment.

The research questions in the present paper were structured according to these three levels:

1. Identification by caregivers: How do formal caregivers become aware of changes possibly due to dementia in the persons in their care?
2. Communication of the suspicion: How and to whom are suspected cases of dementia communicated?
3. Diagnostic assessment: How are the diagnostic assessments of people with intellectual disabilities carried out? Which medical and psychological specialists are involved? Which direct and indirect instruments (e.g., neuropsychological tests, scales and questionnaires) are used in the assessment? How many official diagnoses were made following the assessment?

## 2 Methods

### 2.1 Research Design

We conducted an exploratory, multisite interview study as part of a larger project called ‘DigIDe: Digital Tool for Intellectual Disability and Dementia’, financed by the Styrian Chamber of Labour as part of its digitalisation campaign under the ‘Projektfonds Arbeit 4.0’ initiative. Our research entailed carrying out 30 interviews with formal caregivers, including nurses, care assistants, social assistants and pedagogues (International Standard Classification of Education Level [ISCED] 3 to 7; see Appendix S1) from three care facilities for people with intellectual disabilities and Down syndrome in Styria (Austrian province). All three facilities offer comprehensive services, including residential care and day care. Additionally, each institution offers specialised day care for older people with ID. The three facilities varied considerably in size, ranging from a large provider serving approximately 11 000 individuals, a medium‐sized organisation supporting 700 persons and a smaller institution caring for 200 people.

The interviews were conducted between August 2021 and March 2022 by the first two authors and by two master's students (AH and MT). The students participated in a preparatory training that covered both the interview guide and ethical considerations. They firstly observed the senior researcher conducting one interview. Then, their initial interviews were conducted jointly with the same senior researcher to ensure consistency and adherence to the protocol. To ensure methodological rigour, we followed the Consolidated Criteria for Reporting Qualitative Research (COREQ; Tong et al. 2007) in collecting and reporting our findings. The study adhered to ethical guidelines, including those of the World Medical Association Declaration of Helsinki (2001) and the Ethics Commission of the University of [anonymised name]. Written informed consent for participation in the study and for the publication of anonymised results was obtained from all participants.

### 2.2 Procedure

To recruit participants, we used purposive sampling. A list of potential participants was provided by the cooperating care institutions based on the inclusion criteria communicated by the research team. Interviewee eligibility required at least 2 years of experience as a formal caregiver for people with intellectual disabilities, 1 year of experience working with people with intellectual disabilities and dementia and sufficient knowledge of the German language to participate in the interview. Those willing to participate were contacted via phone or e‐mail to provide further information regarding the objectives and scope of the study and to schedule appointments. Two individuals did not respond to the invitation.

Participants were given the option of conducting the interviews in person or digitally. Twenty‐nine interviews were conducted in person at the participants' workplaces, and one interview was carried out online. The participants gave their written formal consent to the recording and transcription of the interviews, and to the anonymised publication of the results. The interviews lasted between 20 and 100 min (M = 60) and were audio‐recorded using a smartphone. All interview data were fully anonymised and pseudonymised prior to analysis. Anonymity was ensured by assigning coded identifiers, consisting of the interviewer's initials and random digits. Any identifiable information (e.g., names, locations or specific roles) was removed from the transcripts, and the quotations of participants are presented in an anonymised form. Audio recordings and transcripts were securely stored on password‐protected institutional servers in accordance with institutional and data protection regulations. Confidentiality and anonymity were maintained throughout the study.

### 2.3 Participants

The final sample included 30 formal caregivers working in day care centres, sheltered workshops or residential facilities. Participants had a variety of backgrounds (International Standard Classification of Education [ISCED] levels 3 to 7; Schneider 2013); their professional experience ranged from 2 to 33 years (M = 16.6; SD = 8.6). Sixteen caregivers had received training on dementia (e.g., validation and sensory activation; see Appendix S1); only in one case was the training on dementia and disability. Additional information and other demographic details are provided in Table 1 and Appendix S1.

**TABLE 1:** Participants' demographic data.

Gender	Number	Percentage
Female	25	83%
Male	5	17%
Age range		
Unknown	1	3%
25–34	3	10%
35–44	12	40%
45–54	11	37%
55–65	3	10%
ISCED level		
Level 3	12	39%
Level 4	12	40%
Level 5	1	3%
Level 7	5	18%

### 2.4 Materials

The interview guide was developed ad hoc after an extensive literature research. It was then discussed within the group of authors and with other master's students and piloted in July 2021 by the second author and the master's students AH and MT. The interview guide includes socio‐demographic data, followed by questions prompting participants to reflect on a specific person in their care who had dementia or was suspected of having dementia. We then explored the procedures they followed after dementia was suspected. This covered whom they informed first (e.g., colleagues, family members or medical professionals) and how their concerns were addressed within the team. When a diagnostic process took place, caregivers were asked to describe whether external professionals were involved to conduct a neuropsychological assessment and which cognitive tests were administered. This part of the interview guide is presented in Appendix S2.

### 2.5 Data Analysis

The interview recordings were transcribed verbatim following the guidelines of Kuckartz (2018) and analysed with the software MAXQDA 2022 (VERBI Software, 2021). The methodology chosen for the data analysis was the ‘structured qualitative content analysis’ by Mayring (2023). This is a systematic approach to analysing qualitative data by organising it into categories based on predefined criteria, thus ensuring transparency and consistency.

The data analysis was conducted by two PhD students who were not involved in data collection, which helped reduce potential interviewer bias. We adopted a combined approach, allowing for both data‐driven insights and theory‐based structuring. We first developed the categories inductively using Holst et al.'s (2018) model and the interview guide as a basis. Subsequently, we applied a deductive approach to refine and expand the category system. Statements that did not directly relate to the research question or the predefined categories were documented but not included in the final category system, as they were beyond the focus of this study. This decision was made to maintain coherence and analytical depth within the thematic framework. The final categories and subcategories are presented in Appendix S3.

## 3 Results

To assess intercoder reliability, two coders independently coded two identical interviews (approximately 7% of the total sample size of 30 interviews), using jointly agreed code definitions. Intercoder agreement was calculated using MAXQDA 2022 (VERBI Software, 2021), which computed a chance‐corrected Kappa coefficient *K* (*RK*) according to Rädiker and Kuckartz (2019). This coefficient adjusts for the probability that two coders would randomly assign the same codes regardless of the actual data content. The analysis was conducted at segment level, evaluating the overlap and agreement of each segment coded by both coders. Intercoder reliability was assessed based on 90% overlap of the coded material between the two coders, yielding a chance‐corrected kappa coefficient of *K* (*RK*) = 0.85, indicating a high level of agreement (Rädiker and Kuckartz 2019). The themes that emerged from the interviews are presented in terms of the three levels of action and are adapted from the three levels of resource model from Holst et al. (2018). We thus have (1) symptom identification by individual caregivers, (2) communication regarding the suspected case and (3) diagnostic assessment.

### 3.1 First Level—Symptom Identification by Individual Caregivers

#### 3.1.1 Distinguishing Dementia From Intellectual Disability and From Ageing

Many caregivers (*n =* 14) reported finding it challenging to differentiate between symptoms of dementia and the behavioural patterns of the pre‐existing intellectual disability, particularly when the person is new to the caregivers in the facility. In such cases, it is difficult to know which skills or behaviours are pre‐existing and which are due to symptoms of dementia. ‘It's difficult (…) in the disability sector, this, this separation of what it really is (…), from the intellectual side and what is, what is really dementia now? (…) That is difficult’ (13AH, 146). Once behavioural anomalies are noticed, it is then difficult to assess whether such changes are to be attributed to dementia or to the normal ageing process: ‘It was unclear which symptoms were due to age‐related decline and which were actually indicative of dementia. Is it just normal ageing, where you become forgetful, or is it really dementia (…)? That's often difficult (…)’ (12AH, 28).

Caregivers who reported no difficulty also stated that a long‐term relationship with the person is fundamental to being able to notice subtle dementia‐related changes in behaviour, skills and routines, as reported in the next paragraph.

#### 3.1.2 Long‐Term Relationship as the Key to Recognising Dementia

The formal caregivers interviewed emphasised that a long‐term, continuous care relationship, characterised by consistency and emotional depth, is essential for recognising and understanding the early signs of dementia in people with intellectual disabilities. Many of the caregivers stated that the persons in their care had resided in the same facility for an extended period. One caregiver illustrated this point by saying: ‘You have to know them well, that's true, yes, that's usually the case with us, because they live here for a long time, […] for years or some for decades’ (9MT, 20). As a result of this long‐term care, caregivers indicated that they were familiar with the person's everyday habits and knew their individual interests, preferences and abilities. They were able to understand the person's individual communication patterns and interpret their behaviour. This enabled them to quickly identify changes in behaviour, cognitive abilities or daily activities, as one caregiver confirmed: ‘Yes, because we have known this person for a long time, we just notice the changes’ (4AH, 22).

However, caregivers also reported that this long‐standing and emotional relationship with the person with intellectual disabilities is also a challenge. Caregivers emphasised that they are very emotionally involved due to the long‐term relationship and find it difficult to come to terms with the changes that occur. They are reluctant to recognise the deterioration in the person's condition: ‘I mean, like I said, when you've had a relationship with a person for so long it might be easier to recognise something, but the long relationship, and I really dare say this now, also gets in the way. Because he [the person] is very close to you and you don't really want to recognise it [the dementia]’ (9AH, 26).

#### 3.1.3 Documentation of Symptoms

Caregivers highlighted the importance of precise written documentation, considering it indispensable for the objective and precise recording of changes in skills and behaviour. Documentation of this kind enables caregivers to substantiate their observations and establish a solid foundation, ensuring that other healthcare professionals as well as managers take their concerns more seriously.

Seven caregivers reported that they had recorded detailed, dated information independently and systematically. Two caregivers also emphasised that they regularly revaluated the documented changes in order to better understand symptom progression. In one case, this documentation had been done together with a colleague: ‘So, my colleague and I […] spent four or five weeks creating a competency profile […], and last week we sat down together and said that he had deteriorated again, so […] we really saw a deterioration there once again’ (5DP, pos. 16). One caregiver noted that if changes persist for more than 6 months, additional steps are taken, such as consulting a specialist.

However, only two caregivers reported using a standardised survey or observational tool to track changes, although they did not know the exact name of the instrument. Another caregiver mentioned that the functional abilities of the person with intellectual disabilities were assessed using a care sheet in accordance with Böhm (1999). The Böhm's model focuses on understanding and addressing the personal biography of each person, particularly of those in care suffering from dementia, by integrating their life history into caregiving practices.

**FIGURE 1:**
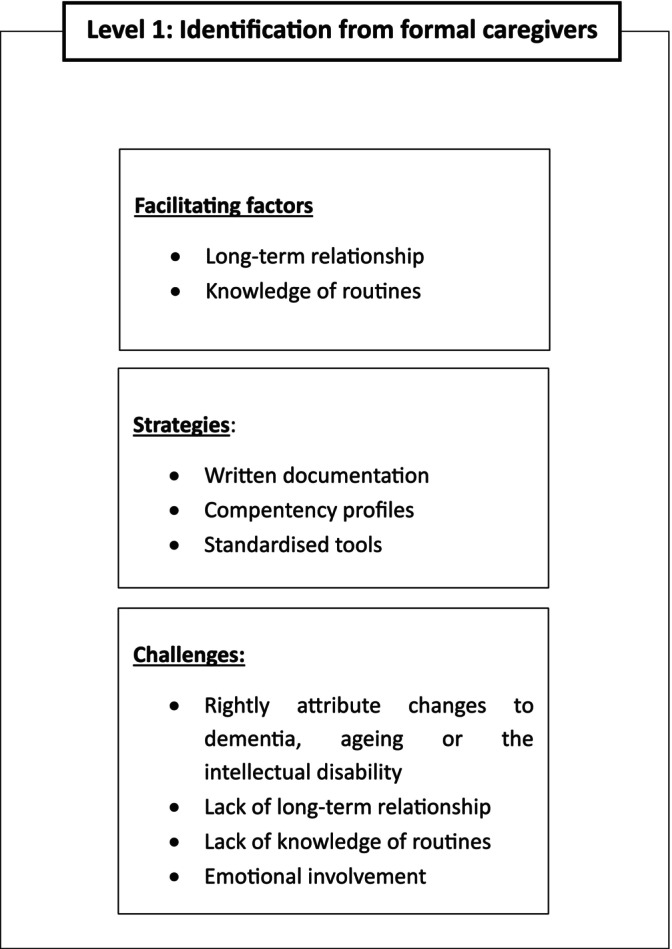
Graphical representation of the themes emerged from the answers to research question 1: ‘How do formal caregivers become aware of changes possibly due to dementia in the persons in their care?’

Figure 1 summarises the results of this section.

### 3.2 Second Level—Communication of Suspected Dementia

#### 3.2.1 Communication With the Team

After caregivers noticed changes possibly attributable to dementia, most of them (*n =* 13) discussed their observations with the whole team. This usually took place during weekly team meetings or care visits attended by all team members and the manager. Some caregivers (*n =* 6) reported that they first discussed their suspicions with individual colleagues rather than with the whole team, while some talked directly to the manager (*n =* 3).

Most caregivers stated that their team agreed that the changes they had observed might be indicative of an onset of dementia. In four cases, the suspicion was rejected by some members of the team and the observed changes were dismissed by colleagues as reflecting an individual's unwillingness to actively participate in everyday life, as laziness or a way of seeking attention. One caregiver described this as follows: ‘Partly a lack of understanding, partly the idea that she wants more help than she needs, […] that she no longer wants to participate, that she wants to be more the centre of attention with these symptoms’ (4MT, 30). Such a dismissive attitude was not only evident among colleagues but also among managers and medical professionals, where suspicions were often not taken seriously. For instance, caregivers have indicated that their observations and suspicions are not always ‘fully accepted by medical professionals’ (1MT, 38). As a result, diagnostic progression came to a halt.

#### 3.2.2 Communication With Relatives

Besides consulting with colleagues within the team and from other departments, caregivers emphasised the importance of engaging with the person's relatives. Nine caregivers indicated that relatives had also noticed similar changes in the behaviour and abilities of the person, as the following examples show: ‘We have talked about it more often, she [the mother] also says that he forgets a lot at home and that it is not like before’ (5DP, 22); ‘So I know (…) that he has also changed at home. That he withdraws and seeks less contact with his parents or sister’ (7AH, 46). One caregiver also noted that dementia was first suspected by the relatives themselves.

At the same time, there are cases where communication with relatives is quite problematic. Some caregivers reported that relatives do not take the observations concerning possible dementia seriously, or they simply reject them. As a result, some family members are resistant to suggestions for intervention, medical clarification and necessary adjustments in care. In other cases, relatives were informed that the person with intellectual disabilities might be developing dementia but showed little interest owing to a strained or difficult relationship with the person. In addition, caregivers also reported cases where there were no relatives left or where contact with them had been completely lost (*n =* 3).

**FIGURE 2:**
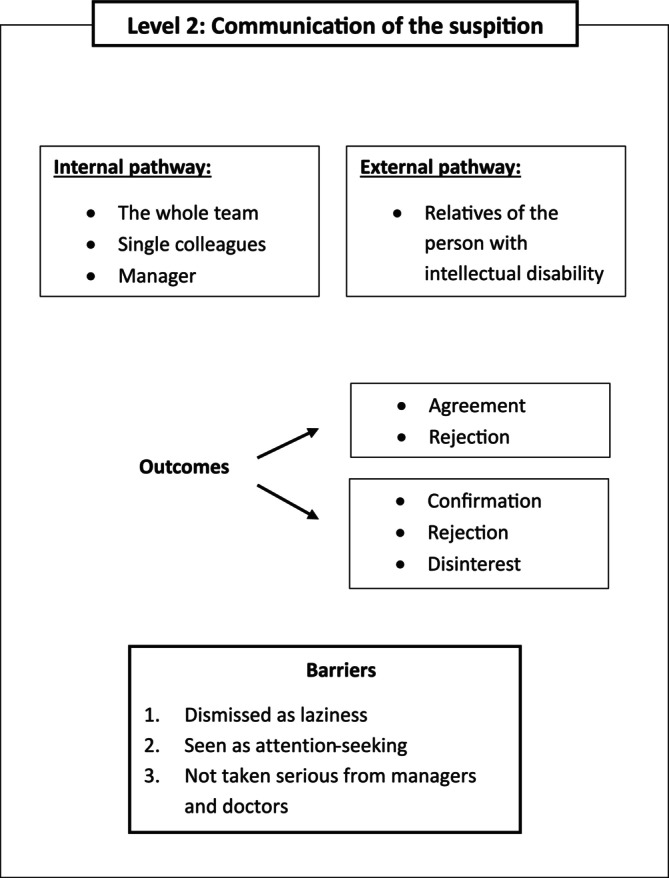
Graphical representation of the themes emerged from the answers to research question 2: ‘How and to whom are suspected cases of dementia communicated?’

Figure 2 provides a summary of the themes that emerged in this section.

### 3.3 Third Level—Diagnostic Assessment

#### 3.3.1 Official Diagnosis

The majority of caregivers (*n =* 19) reported that even when dementia was suspected, no formal diagnosis was made, and no diagnostic procedures were initiated. The remaining 11 caregivers reported that the person in care had been officially diagnosed with dementia by a medical professional or psychologist. Five of them, from the same organisation, reported that tests were conducted directly within their facility by healthcare professionals (doctors or psychologists). In fact, this particular facility has a hospital‐like structure with medical and psychological staff, an uncommon occurrence in Austria. In three of those cases, the testing took place in an environment within the facility that was unfamiliar to the person in care. The remaining six caregivers who reported that a dementia diagnosis had been carried out also stated that the test took place in a setting unfamiliar to the person with intellectual disabilities, either in the family doctor's office or in a hospital.

#### 3.3.2 Diagnostic Clarification

Only three caregivers were able to give precise information about the tests used for the diagnostic assessment and reported that the Mini‐Mental State Examination (MMSE; Folstein et al. 1975) was used. Two other caregivers stated that a test originally developed for people without intellectual disabilities was administered but could not provide further details. However, caregivers were highly critical of the use of such standardised tests for the general population: ‘the tests, I find, are simply not appropriate for our people; they are often really overwhelming. We have many who are illiterate, many who can't do math’ (1MT, 130); ‘it's relatively difficult with such typical tests’ (10AH, 50).

In most cases, the diagnostic clarification was carried out by the family doctor (*n =* 8). However, two caregivers reported that a multi‐professional team of doctors, psychologists and caregivers was involved in the diagnostic process. This was described as lengthy and challenging. One caregiver reported that the diagnostic process, in which a psychologist had been involved, had dragged on for 3 years without a final diagnosis. Another caregiver reported having waited in vain for 2 years for a diagnosis. In some situations, neither neuropsychological tests nor indirect instruments (such as informants' scales) were administered.

**FIGURE 3:**
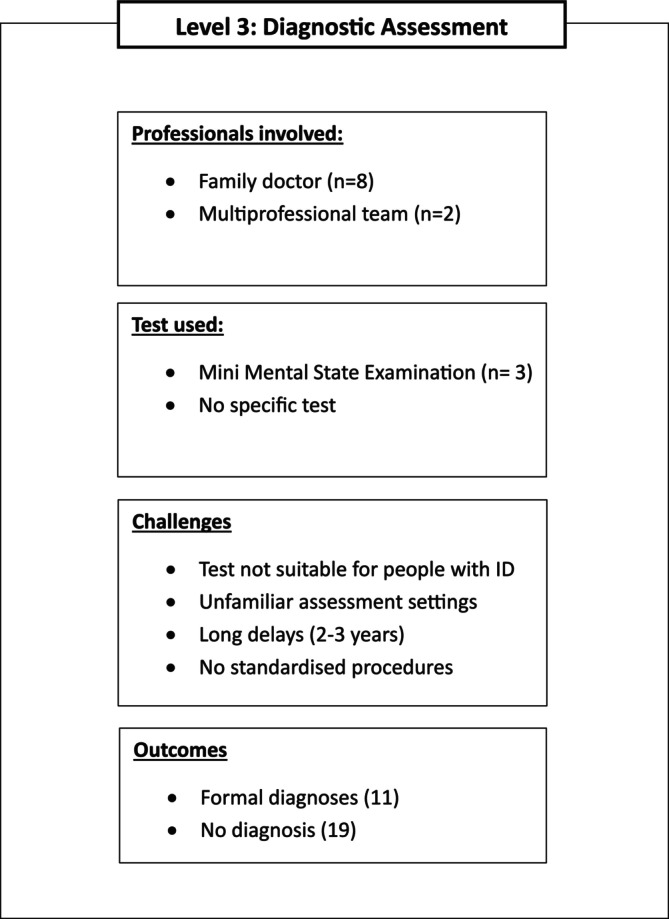
Graphical representation of the themes emerged from the answers to research question 3: ‘How are the diagnostic assessments of people with intellectual disabilities carried out? Which medical and psychological specialists are involved? Which direct and indirect instruments (e.g., neuropsychological tests, scales, questionnaires) are used in the assessment? How many official diagnoses were made following the assessment?’

See Figure 3 for a summary of the themes that emerged in this section.

## 4 Discussion

The present study empirically explored the practices and resources entailed in the process of dementia diagnosis, from initial suspicions to the final diagnosis, within three care facilities in the Austrian province of Styria. We structured our interview and results in accordance with the Holst et al.'s (2018) model of the three levels of resources involved in a diagnostic process: caregivers, team and external professionals (Holst et al. 2018).

Firstly, we deemed it crucial to inquire how formal caregivers notice changes possibly related to dementia, and how they distinguish dementia symptoms from the intellectual disability in the people they care for. The interview responses revealed that long‐term relationships between caregivers and those in their care are essential. They provide caregivers with knowledge about a person's developmental history and their highest level of functioning, used as a reference point for timely detecting dementia‐related changes. In order to optimise the speed and process of referral for a dementia diagnosis, therefore, such long‐term relationships should be encouraged and facilitated in the disability sectors. Unfortunately, the social care sector faces a significantly high staff turnover rate, and staff shortages affect 88% of disability services (Federation of European Social Employers 2023; Schachner et al. 2022). The resulting lack of continuity in caregiver relationships results in several negative consequences for the health, safety and well‐being of the person with an intellectual disability (Friedman 2021). One of these is the ‘overshadowing effect’, which is the misattribution of dementia symptoms to the person's pre‐existing intellectual disability (Mason and Scior 2004). This results in the recognition of dementia symptoms being delayed or met with scepticism among the care team. Such phenomenon was indeed reported by the participants of the present study, whose colleagues often dismissed observed changes or questioned the authenticity of the symptoms perceived, particularly when the person suddenly began to struggle with familiar daily tasks that had previously been performed without difficulty.

To prevent such issues, it is essential to maintain comprehensive written documentation comprising personal data, individual developmental history, skills and preferences and observed changes (NICE Guidelines 2018; Bishop et al. 2015). This can be achieved by using validated, informant‐based dementia assessment instruments, as these can improve the quality and comparability of observations within the team, providing a solid foundation for ascertaining suspected cases of dementia. A review by Zeilinger et al. (2022) provides evidence‐based recommendations for the most suitable and best‐evaluated instruments. Some of these instruments are also available in German. However, in our sample, only a small share of the caregivers interviewed had independently used some form of documentation. Only in some cases did they report having used a standardised tool, but could not report its name. These results highlight how standardised observational and documentation procedures are currently not uniformly established in practice in Austria.

The use of such standardised observational instruments, reducing reliance on caregivers' spontaneous observations, is particularly valuable in settings where specific expertise on dementia in intellectual disabilities is lacking (Acton et al. 2024). Although training on dementia, attended by *n* = 16 of the caregivers interviewed in our sample, undoubtedly provides knowledge of the topic, its usefulness in the context of people with intellectual disabilities is limited, as it is not specifically tailored towards them (Iacono et al. 2014). In fact, dementia manifests differently in people with intellectual disabilities than in the general population (Lautarescu et al. 2017; Strydom et al. 2010; Zeilinger et al. 2022). It is therefore fundamental that caregivers are aware of the specific signs of dementia in this population, as a lack of knowledge about the distinctive symptoms in this group may mean that early, non‐cognitive symptoms, such as apathy and the resultant withdrawal, often go unrecognised (Cleary and Doody 2017; Cipriani et al. 2018; Dekker et al. 2021; Dunning et al. 2025; Furniss and Biswas 2012; Herron and Priest 2013; Whitehouse et al. 2000). Thus, adapting and integrating validated observational instruments into facility documentation systems would provide clinicians and psychologists with more reliable information and enable more accurate and timely diagnostic assessments (Acton et al. 2024; Cipriani et al. 2018; Herron et al. 2020; McCarron and Lawlor 2003; Pendl et al. 2024).

In our sample, only nine out of 30 cases involved a referral to a specialist for diagnostic procedures. These procedures were described as lengthy and, in some cases, inconclusive. In line with previous studies, our results reveal many diagnostic barriers, such as the absence of specialised knowledge and skills among professionals and the inadequacy of diagnostic criteria (Acton et al. 2024; Krinsky‐McHale et al. 2020; Marsack‐Topolewski and Brady 2020; Mcgregor 2025; Zeilinger et al. 2022). Caregivers also reported the use of inadequate diagnostic instruments or settings. In fact, depending on the severity of the intellectual disability, cognitive assessment and physical examination can also be an integral part of diagnostic procedure (Cipriani et al. 2018; NICE 2016). While for profound/severe intellectual disability, informant‐based observational tools are recommended, for less severe cases, direct neuropsychological tests can also be incorporated alongside observational assessments (Dekker et al. 2021). However, such tests should be specifically designed for people with intellectual disabilities to avoid floor effects. In our sample, in *n* = 3 cases, the MMSE (Folstein et al. 1975) was administered as a direct instrument, despite the evidence in the literature that this is an unsuitable tool for this population (Zeilinger et al. 2022).

Additionally, caregivers highlighted the impact of the diagnostic setting, noting that unfamiliar clinical settings can cause stress and negatively influence test performance. Conducting specialist assessments within the residential setting rather than at an external clinic could help mitigate these issues, as demonstrated by the positive experiences reported with on‐site assessments in one of the organisations in the study.

### 4.1 Recommendations for Austria

Our findings reinforce the need in Austria for policy and service development specifically for older adults with intellectual disabilities, a necessity that has been recognised in the literature since the 1970s (Segal 1977; Pendl et al. 2024). Building on our results and on past research, this can be achieved through coordinated effort at three levels. This should involve providing cost‐effective and accessible training opportunities on dementia manifestations, management strategies, and best practices for supporting people with intellectual disabilities for caregivers (Acton et al. 2023; Dekker et al. 2021; Kåhlin et al. 2016; Hughes et al. 2024; Pendl et al. 2024). At the institutional level, measures to reduce caregiver staff turnover should be implemented to ensure continuity of care and long‐term relationships (Acton et al. 2024). At the same time, the introduction of standardised observation instruments, documentation protocols and referral procedures can facilitate timely consultation with dementia specialists (Bishop et al. 2015). Furthermore, dementia‐specific services for older adults with intellectual disabilities staffed by trained specialists should be made available (Duncan et al. 2024).

All this should be supported by a ‘coherent policy to guide service provision and support for this group in either the disability or the aged care sectors’ (Iacono et al. 2014, 521), and by integrating the ageing and disability services (Bickenbach et al. 2012; Heller et al. 2018). Existing Austrian policies and regulations fail in fact to address the specific needs of people with both intellectual disabilities and dementia. The Leistungs‐ und Entgeltverordnung (LEVO‐StBHG 2025), which governs the funding and provision of disability services, does not allocate specific benefits or support measures for people with an intellectual disability and a concurrent dementia diagnosis. As a result, care facilities have to accommodate the complex and relatively new needs of this group without additional financial or structural support.

As long as this theme remains marginal in professional discourse, meaningful progress is unlikely (Denninger 2020; Köhncke 2009). Therefore, the ultimate aim of this study is to shed light on the shortcomings in the care systems for older people with intellectual disabilities, in particular, by emphasising the critical importance of acknowledging dementia—both in policy and in clinical practice. In this sense, the expression ‘name it to tame it’ concisely conveys the core message behind this work: only by recognising and explicitly naming dementia in this population through research, policy changes and diagnostic advancement can we begin to address it effectively.

## 5 Conclusion

This study highlights the critical need for policies and services in Austria that are specifically geared towards older adults with intellectual disabilities. This can be achieved through a coordinated effort in providing caregivers with accessible training on dementia manifestations specific to intellectual disabilities, implementing measures within facilities to reduce staff turnover, alongside standardised observation instruments and referral procedures and establishing dementia‐specific services staffed by trained specialists. All of this requires coherent policies to guide service provision and integrate ageing and disability services. However, current Austrian policies, including the LEVO‐StBHG (2025), fail to address the specific needs of people with both intellectual disabilities and dementia. This leaves care facilities without adequate financial or structural support. Improving the diagnostic process through these coordinated efforts is the first step towards ensuring that everyone can enjoy their right to the highest possible standard of health, regardless of their disabilities (Articles 25 and 26 of the UN CRPD; United Nations 2006).

## Funding

This work was financed and supported by the Styrian Chamber of Labour [*Arbeiterkammer Steiermark*] as part of its digitalisation campaign under the ‘Projektfonds Arbeit 4.0’. This article was published in open access with financial support from the University of Graz.

## Ethics Statement

This study was conducted in accordance with the ethical standards of the ethics board of the University of Graz and the 1964 Declaration of Helsinki. Informed consent was obtained from all participants for the use of anonymised data in scientific research and publication.

## Conflicts of Interest

The authors declare no conflicts of interest.

## Supporting information


**Appendix S1:** Demographic data of the formal caregivers interviewed.
**Appendix S2:** Interview guide translated from German into English.
**Appendix S3:** Coding tree drawn from the areas and subareas of the interview guideline.

## Data Availability

The data supporting the findings of this study are available from the corresponding author upon reasonable request.
