# Supplementary material for: ‘Name It to Tame It’: Dementia Diagnostic Procedure in Austrian Care Facilities for People With Intellectual Disabilities. An Interview Study
Source: J Intellect Disabil Res. 2026 Feb 2;70(5):481–90. doi: 10.1111/jir.70085 (PMC13065438; doi:10.1111/jir.70085)
Supplement: Supplementary file 1 — Appendix S1: Demographic data of the formal caregivers interviewed. Appendix S2: Interview guide translated from German into English. Appendix S3: Coding tree drawn from the areas and subareas of the interview guideline. [file JIR-70-481-s001.docx]

### Appendix A.

Demographic data of the formal caregivers interviewed

| Code | Gender | Age | Education | Work experience  with IDD (Years) | Work experience with dementia and IDD (Years) | Training on dementia |
| --- | --- | --- | --- | --- | --- | --- |
| 1AH | M | Unknown | Training in qualified nursing care (ISCED-Level 3) | 23 | 20 | Instructor for Professor Erwin Böhm's psycho-biographical care model |
| 2AH | M | 58 | Specialized social assistant in the field of disability (ISCED-Level 3) | 5 | 5 |  |
| 3AH | W | 44 | Social pedagogue (ISCED-Level 7) | 16 | 5 | Disability and dementia |
| 4AH | M | 36 | Qualified social assistant and care assistant (ISCED-Level 4) | 7 | 3 |  |
| 5AH | W | 57 | Qualified social assistant (ISCED-Level 4) | 33 | 15 | Böhm training |
| 6AH | M | 36 | Training in qualified nursing care (ISCED-Level 3) | 17 | 3 | Validation |
| 7AH | W | 58 | Assistant for people with disability | 18 | Not known |  |
| 8AH | W | 27 | Specialized social assistant (ISCED-Level 3) | 5 | 5 |  |
| 9AH | W | 50 | Qualified social pedagogue (ISCED-Level 5) | 18 | Not known | At the nursing home, for non-impaired people |
| 10AH | W | 42 | Specialized social assistant in the field of disability (ISCED-Level 3) | 14 | Not known | Communication without words; De-escalation management aid |
| 11AH | W | 43 | Specialized social assistant in the field of disability (ISCED-Level 3) | 22 | 22 | Certified dementia assistant; Kübler-Ross advanced training |
| 12AH | W | 52 | Specialized social assistant in the field of disability (ISCED-Level 3) | 27 | Not known | Internal seminars |
| 13AH | W | 52 | Qualified social assistant in the field of work with people with disabilities ISCED-Level 4) | 25 | 8 | Dementia trainer |
| 2DP | W | 38 | Social pedagogue  (ISCED-Level 7) in the field of work with people with disabilities | 18 | Not known |  |
| 3DP | M | 35 | Qualified social assistant in the field of work with people with disabilities (ISCED-Level 4) | 13 | 3 |  |
| 4DP | W | 44 | Specialized social assistant in the field of disability (ISCED-Level 3) | 3 | 3 |  |
| 5DP | W | 30 | Qualified social assistant in the field of work with people with disabilities (ISCED-Level 4) | 6 | 6 |  |
| 1MT | W | 43 | Qualified social assistant in the field of work with people with disabilities (ISCED-Level 4) | 9 | 9 | Validation training; sensory activation trainer |
| 2MT | W | 37 | Qualified social assistant in the field of work with people with disabilities (ISCED-Level 4) | 14 | 2 | Validation (in house) |
| 3MT | W | 53 | Pedagogue for special needs (ISCED-Level 7) | 26 | 4 |  |
| 4MT | W | 50 | Pedagogue for special needs (ISCED-Level 7) | 25 | 8 |  |
| 5MT | W | 25 | Qualified social assistant in the field of work with people with disabilities (ISCED-Level 4) | 2 | 2 |  |
| 6MT | W | 38 | Social pedagogue  (ISCED-Level 7) | 11 | 5 | Dementia training course |
| 7MT | W | 45 | Qualified social assistant in the field of work with people with disabilities (ISCED-Level 4) | 11 | 11 | Communication without words |
| 8MT | M | 36 | Specialized social assistant in the field of disability (ISCED-Level 3) | 18 | 18 |  |
| 9MT | W | 53 | Training in qualified nursing care (ISCED-Level 3) | 30 | 6 | Böhm training |
| 10MT | W | 49 | Qualified social assistant in the field of work with people with disabilities (ISCED-Level 4) | 7 | Not known |  |
| 11MT | W | 51 | Qualified social assistant in the field of work with people with disabilities (ISCED-Level 4) | 13 | 6 |  |
| 12MT | W | 52 | Qualified social assistant in the field of work with people with disabilities (ISCED-Level 4) | 30 | Not known |  |
| 13MT | F | 52 | Specialized social assistant in the field of disability (ISCED-Level 3) | 21 | Not known |  |

### Appendix B

Interview guide translated from German into English

***Topic***: Interview with caregivers or relatives about perceived symptoms, changes in behaviour as well as abilities and practical daily skills due to dementia.

***Socio-demographic data***

Gender:
Female

Male

Other

Can I ask you about your age?

Can you tell me what training you have had in the disability sector?

How long have you been working with people with intellectual disabilities?

How long have you been working with people with intellectual disabilities and dementia?

Have you attended any further training or additional training, specifically related to dementia? Which ones?

***Suspect of dementia:***

**Now I ask you to think specifically about a person with intellectual impairment and dementia or suspected dementia that you care for. What changes did you observe at the beginning?**

When and why did you suspect dementia in the person you care for?

How were you able to distinguish signs of dementia from the intellectual disability?

What difficulties did you have in distinguishing between the two?

When did you think about dementia?

How did you know it was dementia? Could you elaborate on this / give an example?

***Procedure in case of suspicion:***

How did you proceed after the suspicion?

Which people did you first inform about your suspicion? (e.g.: in the team, caregivers, team management, relatives, family doctor, neuropsychologist)

How was your suspicion dealt with in the team?

What was done first?

How was the diagnostic clarification carried out?

Did a doctor come to the house? Was the person taken to an institution?

Were tests used? If yes, which ones? (e.g. observation, questionnaires, screenings) - Could you elaborate on this/give an example?

***Thank you very much for taking the time.***

###

### Appendix C

Coding tree drawn from the areas and subareas of the interview guideline

| LEVEL | CATEGORY | SUBCATEGORIES |
| --- | --- | --- |
| IDENTIFICATION BY INDIVIDUAL CAREGIVERS | SUSPICION OF DEMENTIA | Through OBSERVATION |
|  |  | Through DOCUMENTATION |
|  |  | Through STANDARDISED INSTRUMENTS |
|  | DIFFERENTIATION ID AND DEMENTIA | Through noticing CHANGES |
|  |  | Through PERSONAL KNOWLEDGE |
|  |  | |
|  |  | |
| (internal) COMMUNICATION | SUSPICION DISCUSSED WITH | THE TEAM |
|  |  | A COLLEAGUE |
|  |  | THE MANAGER |
|  |  | THE PERSONS´ FAMILY |
|  | ANSWER TO THE EXPRESSED SUSPICION | AGREEMENT OF THE SUSPECT |
|  |  | DISAGREEMENT |
|  |  | |
| DIAGNOSTIC ASSESSMENT | DIAGNOSTIC PROCESS | DURATION |
|  |  | RESULT OF THE DIAGNOSIS |
|  |  | PROFESSIONALS INVOLVED |
|  | ASSESSMENT | NEUROPSYCHOLOGICAL TEST USED |
|  |  | SETTING |
